# IL-6 and NFE2L2: A putative role for the hepatoprotective effect of *N*. *Sativa, P*. *Ginseng* and *C. Sempervirens* in AFB-1 induced hepatocellular carcinoma in rats

**DOI:** 10.1016/j.toxrep.2019.05.008

**Published:** 2019-05-21

**Authors:** Nora M. Aborehab, Nermien E. Waly

**Affiliations:** 1 Department of Biochemistry, Faculty of Pharmacy, October University for Modern Sciences and Arts (MSA University), 6th October Giza 12611, Egypt; 2 Department of Physiology, Faculty of Medicine, Helwan University, Cairo, 11795, Egypt,; 3 Department of Medical Education, Creighton School of Medicine, Omaha, NE, zip 68175, USA

**Keywords:** HCC, NFE2L2, N. Sativa, P. Ginseng, C. Sempervirens, IL-6

## Abstract

In this study, we investigated possible hepato-protective effects of *N. Sativa*, *P. Ginseng*, and *C. Sempervirens in* Aflatoxin B1 (AFB-1) induced hepatocellular carcinoma rat model.

Fifty-four male albino rats were randomly assigned to experimental groups. Alcoholic extracts of aforementioned herbs were administered orally for 28 days at different doses. IL-6, hs-CRP, MDA, SOD and NFE2L2 were determined using ELISA. Histopathological changes in treated groups were examined.

Herbal treatment significantly reduced IL-6, hs-CRP, and MDA (*P* < 0.001) whereas it significantly increased SOD (p < 0.001). *C. Sempervirens* 600 and *N. Sativa* 1000 increased NFE2L2 level compared to *P. Ginseng* 500 group (*P* value<0.01). Histopathological evaluation of treated groups showed different grades of healing of the liver.

This study confirms a beneficial hepatoprotective effect for aforementioned herbal extracts orally administered in rat model of AFB1 induced HCC. This effect is putatively mediated via modulation of inflammatory cytokines as well as amelioration of oxidative stress.

## Highlights

• *P. Ginseng* showed a prominent prophylactic effect in AFB-1 induced rat model.
• Hepatoprotective effects of extracts possibly mediated via IL-6, hs-CRP, SOD, NFE2L2.
• NFE2L2 play a pivotal role in this hepatoprotective effect of herbal extracts.

## Graphical abstract

**Figure:**
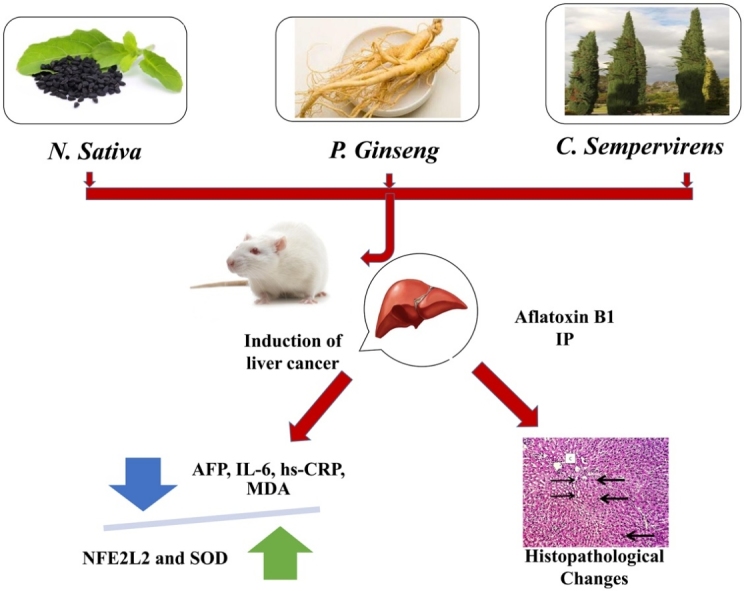

## 1 Introduction

Hepatocellular carcinoma (HCC) is considered to be a primary cause of cancer related deaths globally [[1], [2], [3]]. There may rise of the incidence of HCC in Egypt due to several risk factors e.g. hepatitis C infection [4,5]. With the highest prevalence of HCV infection worldwide, finding alternative as well as preventive therapy for HCC is of a great concern to researchers and oncologists both in Egypt and globally [3,6,7]. Herbal medicine poses a potential complementary and alternative medicine for many diseases including HCC [8,9].

*Nigella Sativa (N. Sativa)* also known as black or Blessings seed is a common food condiment in the middle east and has been widely used by several cultures as a treatment of various diseases [10]. *N. Sativa* was reported to have anti-inflammatory and anti-oxidant effect [[10], [11], [12]]. Its anti-cancer and chemotherapeutic actions both in vitro and in vivo animal cancer models have also been reported [13,14]. Not only that, but *N. Sativa* has also been shown to have protective effects against chemotherapeutic agent’s side effects e.g. cardio- and nephrotoxicity [[15], [16], [17]].

*Panax Ginseng (P. Ginseng)* is a Chinese herb that has been used commonly in medicine. Studies have shown Ginseng family members of herbs have potential roles as anticancer agents [18,19]. An anti-angiogenic saponin; Ginsenoside Rg3 extracted from ginseng was used for the treatment of HCC tumors in rats via reducing metastasis and promoting more survival [20]. Another study has shown that fermented Ginseng (FG) had hepatoprotective effect in liver cancer rat model [21]. In patients of epithelial ovarian cancer (EOC), who received chemotherapy, red ginseng reduced genotoxicity and improved quality of life [19]. Another study illustrated the mechanism of action of ginsenosides in Alzheimer’s disease and the effect of different factors on the yield of extractable materials [22,23].{Razgonova, 2019 #62}{Razgonova, 2019 #62}{Razgonova, 2019 #62}{Razgonova, 2019 #62}

C*upressus Sempervirens (C. Sempervirens)* commonly known as American yellow jasmine was reported to have potential anticancer effects both in vitro and in vivo [24,25]. It is a native herb in north Africa that possesses several therapeutic actions [26]. Its extract was shown to inhibit cancer cell proliferation in mouse skin carcinoma model [27].

Studies have shown that inflammation and oxidative stress are key components in the pathophysiology of HCC [28,29]. In fact inflammatory condition and pro-oxidative state may synergistically act to enhance tumor progression [30]. Inflammatory cytokines e.g. IL-6 plays a central role in the pathogenesis of HCC [31]. Targeting antioxidant enzymes and inflammatory pathways, pharmacologically, poses a logical potential strategy to control tumor-genesis in HCC models [30,32].

Nuclear factor (erythroid-derived 2)-like 2, also known as NFE2L2 or Nrf2 plays an important role in activating antioxidant response in the cell for its protection from exogenous and endogenous insults; moreover, it is thought to be a cytoprotective transcription factor and the main regulator of the survival of the cell [33].

The anti-tumor mechanism of action of these three aforementioned herbs is an active area of research and yet to be elaborated. In this study, we investigated possible hepato-protective effects of *N. Sativa*, *P. Ginseng*, and *C. Sempervirens in* Aflatoxin B1 (AFB-1) induced hepatocellular carcinoma rat model. We hypothesized that the mechanism of this hepato-protective action of these herbs act is putatively mediated via anti-oxidant and anti-inflammatory effects.

## 2 Material and Methods

### 2.1 Animals

Fifty-four male albino rats, 200 ± 20 grams (gm) at the start of the experiments were used. Animals were randomly assigned to experimental groups. Four rats (maximum) were housed per cage (size 26 × 41 cm) and placed in the experimental room for acclimatization 24 hours before any procedure performed. Animals were fed with standard laboratory diet, tap water ad libitum, and kept in an air-conditioned animal room at 23 ± 1 °C with a 12 h light/dark cycle.

Animal care and handling was performed in conformance with approved protocols of MSA University and Egyptian Community guidelines for animal care. MSA Faculty of Pharmacy Research Ethics Committee approved the study

### 2.2 Chemicals and drugs

• **Aflatoxin** B1 (AFB1) was purchased from Sigma-Aldrich Chemicals Co., Egypt, dissolved in 0.9% saline and administered intraperitonealy (i.p.) at dose 150 μg/kg/day for 3 days to induce HCC according to methodology described in *Kim et al., 2011* [34,35].
• **Silymarin** (Legalon™) was purchased from Rottapharm Madaus, Egypt, was dissolved in 0.25% Carboxy Methyl Cellulose (CMC) and administered orally.
• **Alcoholic extracts of*N. Sativa*seeds*P. Ginseng*roots and C*. Sempervirens*** leaves, were purchased from United group Pharma, (UGPharma), Egypt. Extracts were dissolved in Carboxy Methyl Cellulose (CMC) (0.25%) and administered orally using the rat feeding tube [36].

### 2.3 Experimental groups

Experimental doses of all herbs were selected based on previous studies and within non-toxic range [26,37,38]. Rats were randomly allocated into nine groups of six animals each.

• **Group 1 (control):** control group injected (i.p.) by 100 μl saline.
• **Group 2 (HCC):** rats were injected (i.p.) with AFB1 at dose 150 μg/kg/day for 3 days.
• **Group 3 (*C. Sempervirens*300):** rats received *C*. *Sempervirens* extract 300 mg/kg/day, orally for 28 days prior to AFB1 treatment.
• **Group 4 (*C. Sempervirens*600):** rats received *C*. *Sempervirens* extract 600 mg/kg/day, orally for 28 days prior to AFB1 treatment.
• **Group 5 (*P. Ginseng*250):** rats received *P. Ginseng* extract 250 mg/kg/day, orally for 28 days prior to AFB1 treatment.
• **Group 6 (*P. Ginseng*500):** rats received *P. Ginseng* extract 500 mg/kg/day, orally for 28 days prior to AFB1 treatment.
• **Group 7 (*N. Sativa*500):** rats received *N. Sativa* extract 500 mg/kg/day, orally for 28 days prior to AFB1 treatment.
• **Group 8 (*N. Sativa*1000):** rats received *N. Sativa* extract 1000 mg/kg/day, orally for 28 days prior to AFB1 treatment.
• **Group 9 (Silymarin):** rats received silymarin 30 mg/kg/day, orally for 28 days prior to AFB1 treatment.

### 2.4 Blood samples and biochemical analysis

#### 2.4.1 Preparation of blood samples

At the end of the study, rats were fasted overnight, anesthetized with thiopental sodium (50 mg/kg) [39] and blood samples were collected (5 ml per rat). Blood samples were centrifuged at 3000 rpm for 15 min after 30 minutes of collection and stored at –80 ^0^C until analyzed. Serum Alpha-fetoprotein (AFP), Interleukin-6 (IL-6) and high sensitive C-reactive protein (hs-CRP) were determined using the rat enzyme immunoassay kits.

#### 2.4.2 Preparation of liver samples

Animals were euthanized by cervical dislocation at 24 hours of AFB1 injection, and then liver was rapidly removed from each rat. Each liver was divided into two parts. The first part was fixed in formalin-saline for 48 hours for histopathological study. The second part was homogenized, using glass homogenizer (Universal Lab. Aid MPW-309, mechanika precyzyjna, Poland), with 5 mL phosphate buffer saline (PBS) then centrifuged using cooling ultra-centrifuge. The homogenate was divided into three aliquots for measuring total protein, malondialdehyde (MDA) and superoxide dismutase (SOD). Another part of the liver was homogenized with 5 mL phosphate buffer saline (PBS), using glass homogenizer and was subjected to nuclear extraction protocol according to methodology of LSBio^TM^ DNA-Binding ELISA Kit contains the necessary buffers and inhibitors for nuclear extraction from cells for measuring Nuclear Factor Erythroid Derived 2 Like 2 Protein (NFE2L2).

#### 2.4.3 Biochemical analysis

Serum analysis was performed to measure AFP, IL-6 and hs-CRP levels using rat respective enzyme immunoassay kits (Wuhan Fine Biological Technology Co., Ltd, China, Immuno-Biological Laboratories, Inc. (IBL-America), Wuhan EIAAB science Co, Ltd, China). Liver MDA and SOD were measured using colorimetric method according to manufacturer instructions using commercial kits purchased from Biodiagnostic, Egypt, while Liver NFE2L2 was measured using rat immunoassay kits purchased from Cloud Clone Corp, USA.

### 2.5 Histopathological Examination

At the end of the study, livers were harvested, as mentioned previously. Specimens were fixed in 10% formalin and then liver tissues were decalcified in nitric oxide for 4 days, routinely processed and embedded in paraffin. Five microns sections were cut and stained with hematoxylin and Eosin (H&E).

### 2.6 Statistical analyses

All data were expressed as mean ± SEM and analyzed using Prism^®^ program version 6. For all parameters, comparisons among groups were carried out using one-way analysis of variance (ANOVA) followed by Bonferroni’s multiple comparisons test. All *P* values reported are two-tailed and *P*<0.05 were considered significance.

## 3 Results

### 3.1 Effect of *C. Sempervirens*, *P. Ginseng* and *N. Sativa* extracts on liver enzymes level

Mean serum level of liver enzymes were significantly increased in AFB1 induced HCC group compared to the control group; P value was < 0.0001. The mean serum levels of ALT & AST were significantly reduced in *C. Sempervirens* 300, *C. Sempervirens* 600, *P. Ginseng* 250, *P. Ginseng* 500, *N. Sativa* 500, *N. Sativa* 1000 and silymarin groups compared to AFB1 induced HCC group (P value < 0.0001). Non-significant difference was found between 3 herbs in reducing liver enzymes (Table 1).

**Table 1:** Effect of *N. Sativa*, *P. Ginseng* and *C. Sempervirens* extracts on serum ALT & AST levels.

Groups	ALT (U/L)	AST (U/L)
**Control**	21 ± 0.73	21.5 ± 0.76
**HCC**	146 ± 3.36 ^a^	155.8 ± 3.42 ^a^
***C. Sempervirens* 300**	59.3 ± 3.5 ^ab^	67.1 ± 2.79 ^ab^
***C. Sempervirens* 600**	32 ± 3.1 ^ab^	38.3 ± 2.30 ^ab^
***P.Ginseng* 250**	55.1 ± 2.31 ^ab^	67 ± 1.23 ^ab^
***P.Ginseng* 500**	29.6 ± 0.88 ^ab^	38.8 ± 1.13 ^ab^
***N.Sativa* 500**	60.5 ± 4.15 ^ab^	68 ± 2.35 ^ab^
***N.Sativa* 1000**	31 ± 1.53 ^ab^	36.6 ± 1.83 ^ab^
**Silymarin**	34.8 ± 1.19 ^ab^	40.8 ± 1.13 ^ab^

Results were expressed as mean ± SEM and analyzed using one-way ANOVA followed by Bonferroni’s post hoc test, a: significant from control at *P* < 0.0001, b: significant from AFB1 induced HCC group at *P* < 0.0001

### 3.2 Effect of *N. Sativa*, *P. Ginseng* and *C. Sempervirens* extracts on serum Alpha-fetoprotein (AFP) level

**Fig. 1:**
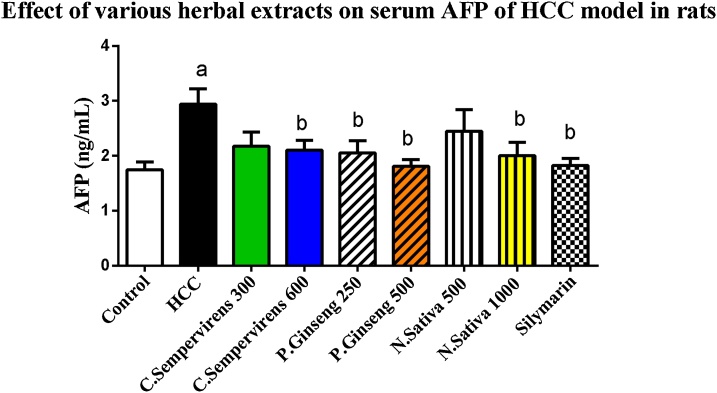
Serum level of Alpha-fetoprotein (ng/mL) in the experimental groups. *N. Sativa*, *P. Ginseng* and *C. Sempervirens* extracts reduced serum level of AFP in the liver cancer rats after 1 month of prophylaxis. Results were expressed as mean ± SEM and analyzed using one-way ANOVA followed by Bonferroni’s post hoc test, a: significant from control at *P* < 0.05, b: significant from AFB1 induced HCC group at *P* < 0.05

As shown in Fig. 1, the mean serum level of AFP was significantly increased in AFB1 induced HCC group compared to the control group, P value was < 0.05. The mean serum level of AFP was significantly reduced in *C. Sempervirens* 600, *P. Ginseng* 250, 500, *N. Sativa* 1000 and silymarin groups compared to AFB1 induced HCC group (P value < 0.05).

### 3.3 Effect of *N. Sativa*, *P. Ginseng* and *C. Sempervirens* extracts on serum Interleukin-6 (IL-6) and high sensitive C-reactive protein (hs-CRP)

**Fig. 2:**
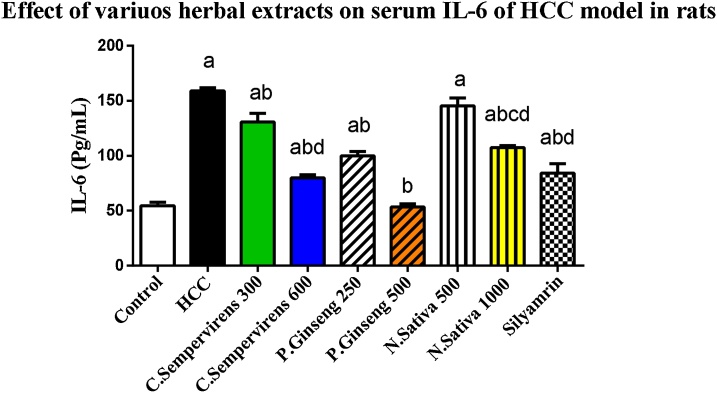
Serum level of IL-6 (Pg/mL) in the experimental groups. *N. Sativa*, *P. Ginseng* and *C. Sempervirens* extracts reduced serum level of IL-6 in the liver cancer rats after 1 month of prophylaxis. Results were expressed as mean ± SEM and analyzed using one-way ANOVA followed by Bonferroni’s post hoc test, a: significant from control at *P* < 0.0001, b: significant from AFB1 induced HCC group at *P* < 0.0001, c: significant from *C. Sempervirens* 600 at *P* < 0.0001, d: significant from *P. Ginseng* 500 at *P* < 0.0001

Fig. 2 shows that, the mean serum level of IL-6 was significantly increased in AFB1 induced HCC group compared to the control group, *P* value was < 0.0001, the mean serum level of IL-6 was significantly reduced in *C. Sempervirens* 300, 600, *P. Ginseng* 250, 500, *N. Sativa* 1000 and silymarin groups compared to AFB1 induced HCC group (*P* value < 0.0001), *P. Ginseng* 500 reduced IL-6 level compared to *C. Sempervirens* 600, *N. Sativa* 1000 and Silymarin groups (*P* value was < 0.0001) also, non-significant difference was found between control and *P. Ginseng* 500 group in serum level of IL-6

**Fig. 3:**
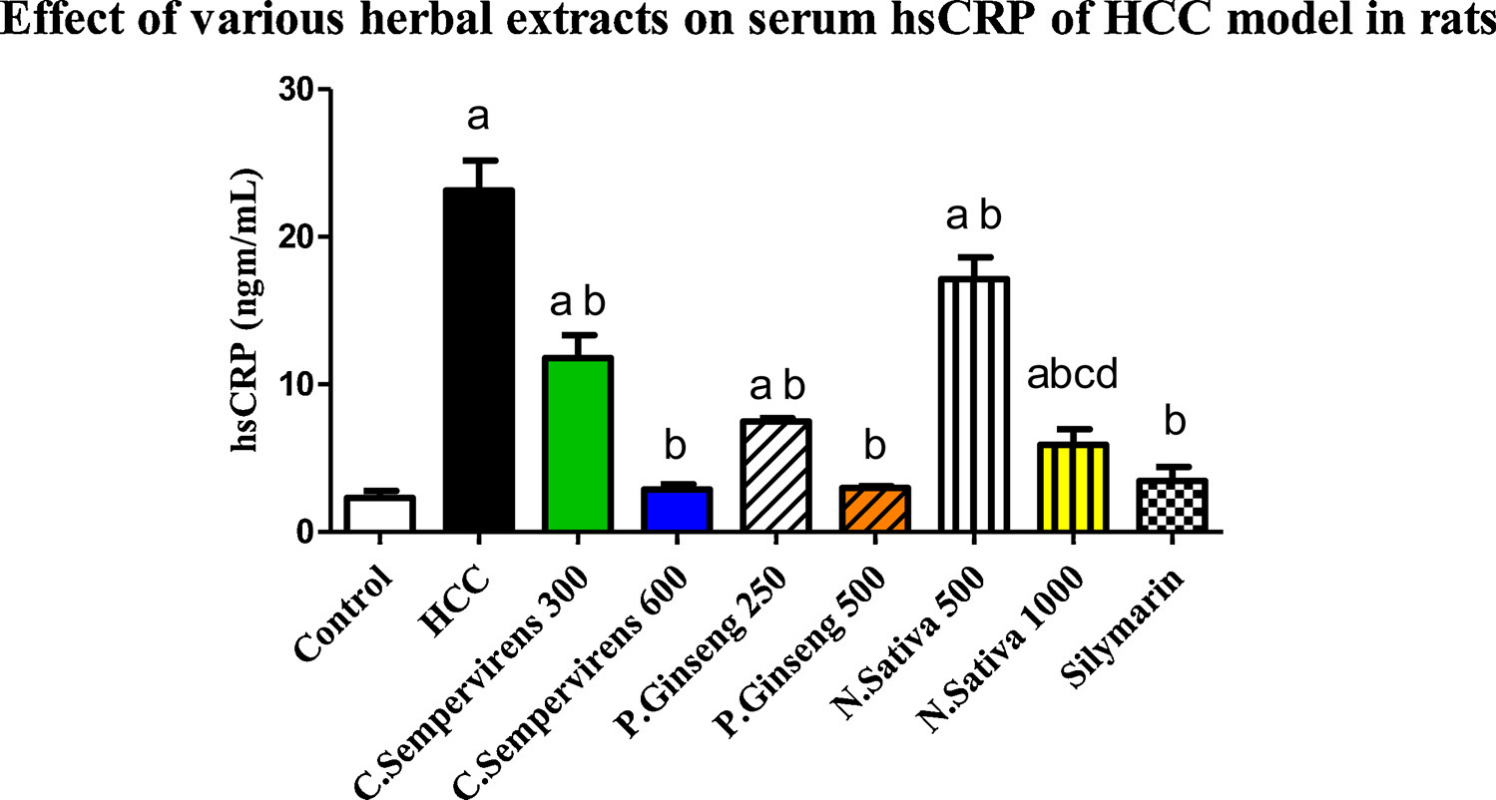
Serum level of hs-CRP (ng/mL) in the experimental groups. *N. Sativa*, *P. Ginseng* and *C. Sempervirens* extracts reduced serum level of hs-CRP in the liver cancer rats after 1 month of prophylaxis. Results were expressed as mean ± SEM and analyzed using one-way ANOVA followed by Bonferroni’s post hoc test, a: significant from control at *P* < 0.01, b: significant from AFB1 induced HCC group at *P* < 0.001, c: significant from *C. Sempervirens* 600 at *P* < 0.05, d: significant from *P. Ginseng* 500 at *P* < 0.05.

Fig. 3 shows that the mean serum level of hs-CRP was significantly increased in AFB1 induced HCC group compared to the control group, *P* value was < 0.01. The mean serum level of hs-CRP was significantly reduced in *C. Sempervirens* 300, 600, *P. Ginseng* 250, 500, *N. Sativa* 500, 1000 and silymarin groups compared to AFB1 induced HCC group (P value < 0.02). *C. Sempervirens* 600 and *P. Ginseng* 500 showed a better result compared to *N. Sativa* 1000 in reducing serum hs-CRP levels. Non-significant difference was found between silymarin group and *C. Sempervirens 600* and *P. Ginseng 500* in the serum level of hs-CRP

### 3.4 Effect of *N. Sativa*, *P. Ginseng* and *C. Sempervirens* extracts on tissue Malondialdehyde (MDA) and Superoxide dismutase (SOD)

**Fig. 4:**
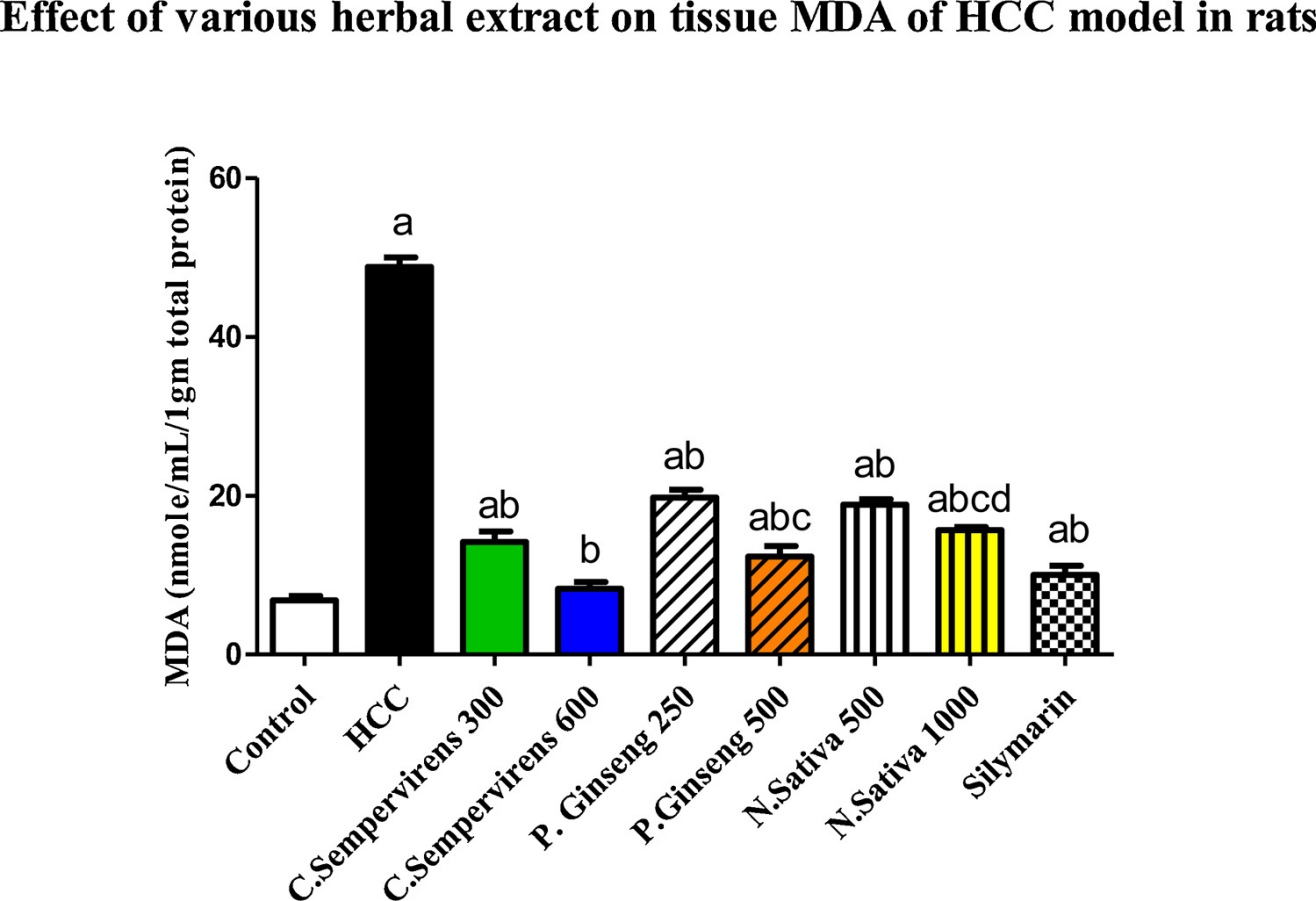
Tissue level of MDA (nmole/mL/1gm total protein) in the experimental groups. *N. Sativa*, *P. Ginseng* and *C. Sempervirens* extracts reduced tissue level of MDA in the liver cancer rats after 1 month of prophylaxis. Results were expressed as mean ± SEM and analyzed using one-way ANOVA followed by Bonferroni’s post hoc test, a: significant from control at *P* < 0.0001, b: significant from AFB1 induced HCC group at *P* < 0.0001, c: significant from *C. Sempervirens* 600 at *P* < 0.05, d: significant from *P. Ginseng* 500 at *P* < 0.05.

The mean tissue level of MDA was significantly increased in AFB1 induced HCC group compared to the control group, *P* value was < 0.0001, the mean tissue level of MDA was significantly reduced in *C. Sempervirens* 300, 600, *P. Ginseng* 250, 500, *N. Sativa* 500, 1000 and silymarin groups compared to AFB1 induced HCC group (*P* value < 0.0001), *C. Sempervirens* 600 reduced MDA level compared to *P. Ginseng* 500, *N. Sativa* 1000 groups even to control group (*P* value was < 0.03) also, Silymarin showed a good response when compared to *C. Sempervirens* 600 and *P. Ginseng* 500 groups (Fig. 4)

**Fig. 5:**
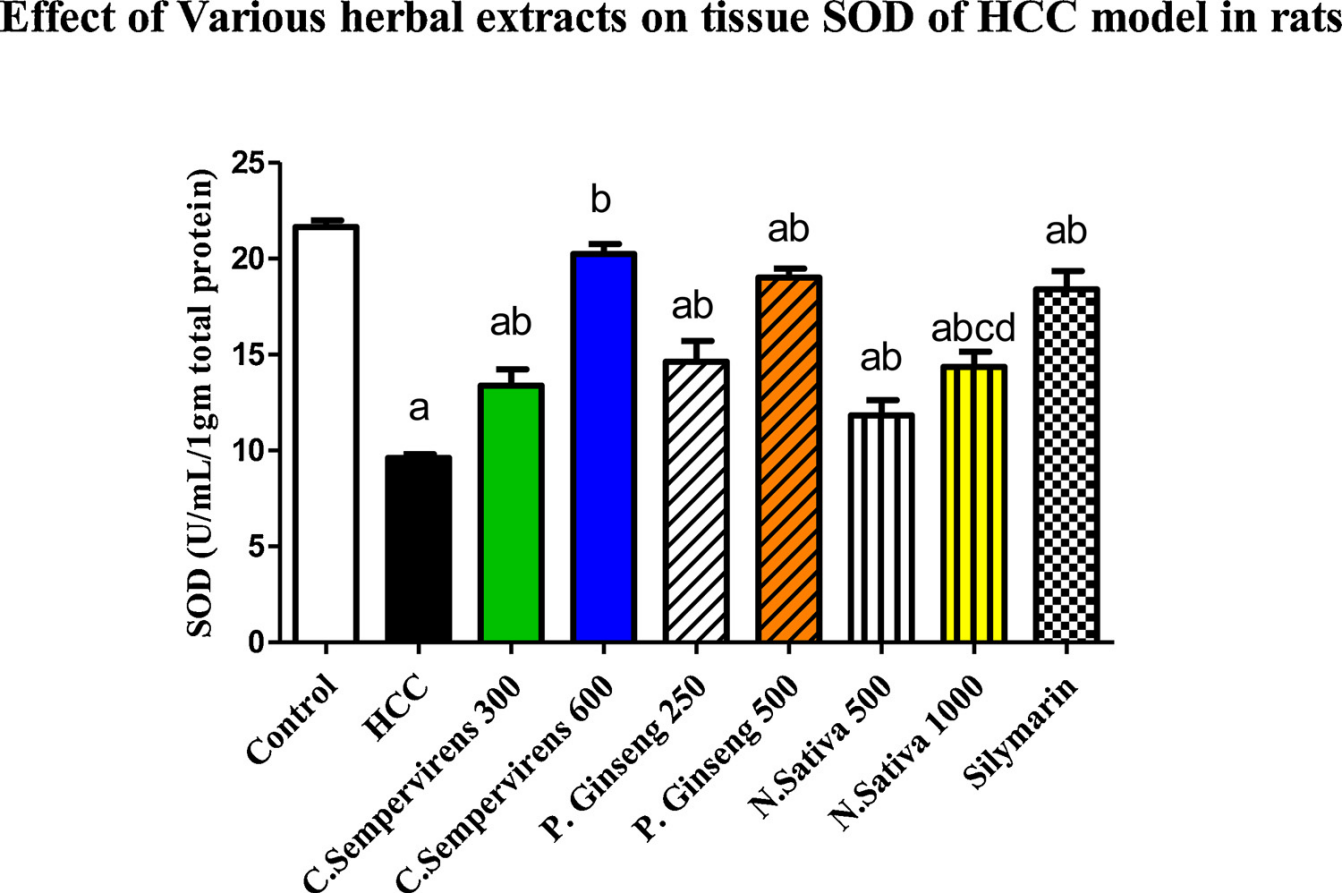
Tissue level of SOD (U/mL/1 gm total protein) in the experimental groups. *N. Sativa*, *P. Ginseng* and *C. Sempervirens* extracts reduced tissue level of SOD in the liver cancer rats after 1 month of prophylaxis. Results were expressed as mean ± SEM and analyzed using one-way ANOVA followed by Bonferroni’s post hoc test, a: significant from control at *P* < 0.0001, b: significant from AFB1 induced HCC group at *P* < 0.0001, c: significant from *C. Sempervirens* 600 at *P* < 0.0001, d: significant from *P. Ginseng* 500 at *P* < 0.0001

The mean tissue level of SOD was significantly decreased in AFB1 induced HCC group compared to the control group, *P* value was < 0.0001, the mean tissue level of SOD was significantly increased in *C. Sempervirens* 300, 600, *P. Ginseng* 250, 500, *N. Sativa* 500, 1000 and silymarin groups compared to AFB1 induced HCC group (*P* value < 0.0001). Non-significant difference between *C. Sempervirens* 600 and *P. Ginseng* 500 groups in tissue level of SOD (Fig. 5), on the other hand *C. Sempervirens* 600 and *P. Ginseng* 500 raised the SOD level compared to *N. Sativa* 1000 group, *P* value was < 0.0001

### 3.5 Effect of *N. Sativa*, *P. Ginseng* and *C. Sempervirens* extracts on tissue Rat Nuclear Factor Erythroid Derived 2 Like 2 Protein (NFE2L2)

**Fig. 6:**
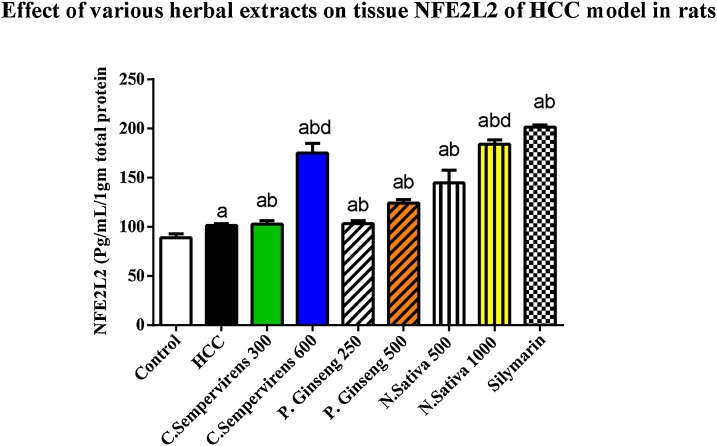
Tissue level of NFE2L2 (U/mL/1gm total protein) in the experimental groups. *N. Sativa*, *P. Ginseng* and *C. Sempervirens* extracts raised tissue level of NFE2L2 in the liver cancer rats after 1 month of prophylaxis. Results were expressed as mean ± SEM and analyzed using one-way ANOVA followed by Bonferroni’s post hoc test, a: significant from control at *P* < 0.0001, b: significant from AFB1 induced HCC group at *P* < 0.0001, d: significant from *P. Ginseng* 500 at *P* < 0.01

The mean tissue level of NFE2L2 was significantly increased in AFB1 induced HCC group compared to the control group, *P* value was < 0.0001, the mean tissue level of NFE2L2 was significantly raised in *C. Sempervirens* 300, 600, *P. Ginseng* 250, 500, *N. Sativa* 500, 1000 and silymarin groups compared to AFB1 induced HCC group (*P* value < 0.0001), *C. Sempervirens* 600 and *N. Sativa* 1000 increased NFE2L2 level compared to *P. Ginseng* 500 group (*P* value was < 0.01) also, Silymarin showed a good response when compared to *C. Sempervirens* 600 and *P. Ginseng* 500 groups. Significant difference was found between the N. sativa 500 and 1000 groups (*P* value was < 0.01) (Fig. 6)

### 3.6 Histopathological effects of *N. Sativa*, *P. Ginseng* and *C. Sempervirens* extracts

**Fig. 7:**
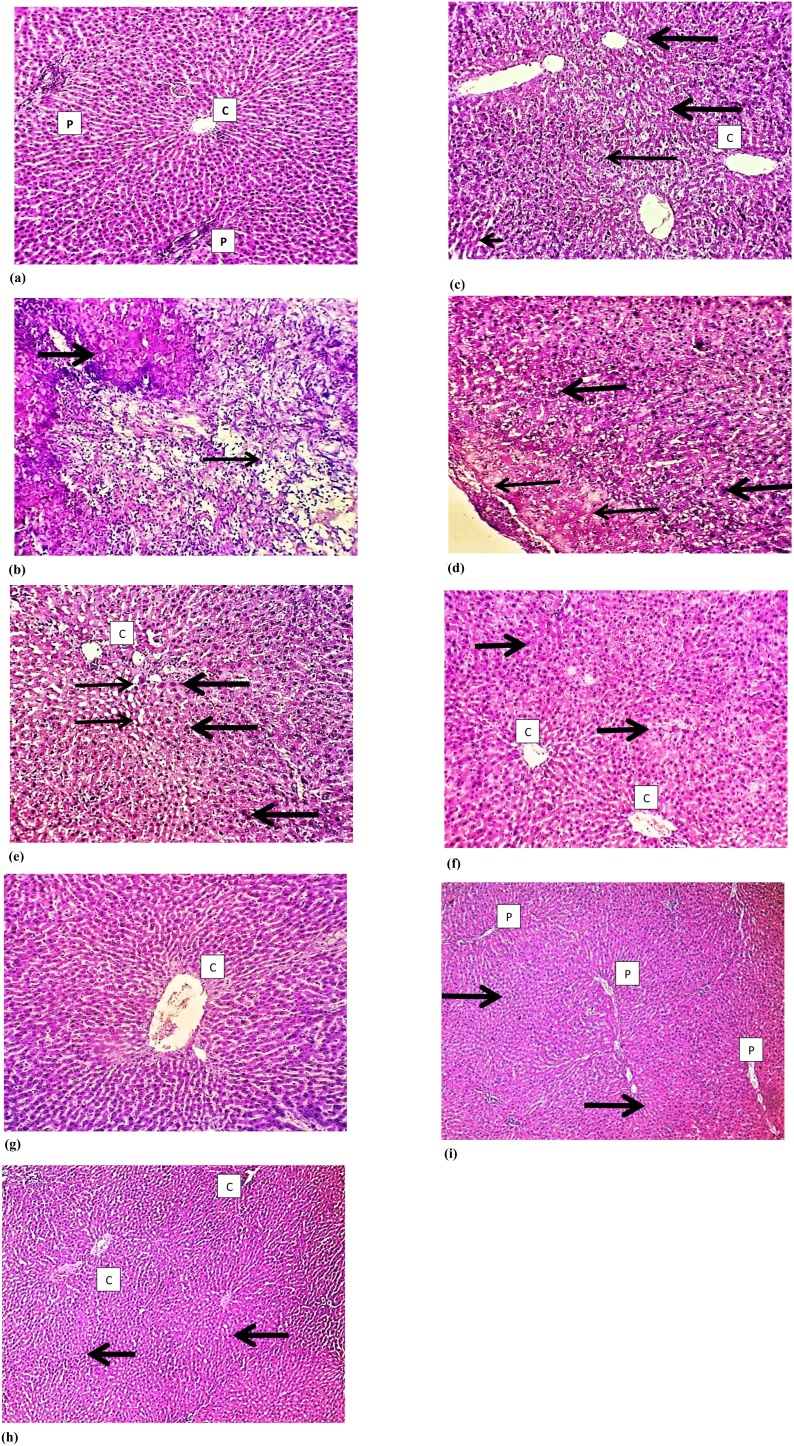
Histological examination of the liver tissue of all experimental groups using Hematoxylin and Eosin (H&E) staining: a) illustrates control group sections that shows normal hepatocytes and preserved hepatic lobular architecture, with regular central veins (C) and unremarkable portal tracts (P). b) Illustrates Aflatoxin group section that shows wide neoplastic transformation of the hepatocytes, with increased N/C ratio, nuclear hyperchromasia and irregular nuclear membranes. c) Illustrates Silymarin treated group section that shows preservation of the lobular architecture, subserosal hepatocyte dysplastic changes is focally evident, however with no frank neoplastic transformation. d) Illustrates *N*. *Sativa* group sections that shows evident preservation of the lobular architecture, minimal hepatocyte dysplastic changes is focally evident (thick arrow), however with no neoplastic transformation. e) and f) illustrates C. *Sempervirens and P*. *Ginseng* group sections that shows evident preservation of the lobular architecture with unremarkable hepatocyte dysplastic changes and no neoplastic transformation. Magnification 10 × .

Histological examination of the liver tissue of all experimental groups using Hematoxylin and Eosin (H&E) staining revealed that normal hepatocytes and preserved hepatic lobular architecture, with regular central veins (C) and unremarkable portal tracts (P) of the liver of the control group. Sections show wide neoplastic transformation of the hepatocytes, with increased N/C ratio, nuclear hyperchromasia and irregular nuclear membranes in Aflatoxin group. In Silymarin treated group sections showed preservation of the lobular architecture, subserosal hepatocyte dysplastic changes is focally evident with no frank neoplastic transformation. Examination *N*. *Sativa* group *P. Ginseng* and *C. Sempervirens* treated sections showed evident preservation of the lobular architecture, minimal hepatocyte dysplastic changes are focally evident with no neoplastic transformation (Fig. 7).

## 4 Discussion

This study clearly shows and confirms a hepatoprotective role for *N. Sativa*, *P. Ginseng* and *C. Sempervirens* extracts in AFB1 induced HCC rat model evidenced by reduction of liver enzymes (AST and ALT), tumor marker (AFP) and histopathological observations. This hepatoprotective effect is possibly mediated via antioxidant and anti-inflammatory effects as *N. Sativa*, *P. Ginseng* and *C. Sempervirens* extracts, orally administered for about a month, clearly improved inflammatory (IL-6 and hs-CRP) as well as antioxidant (MDA and SOD) biochemical markers. These effects were comparable to the effects of Silymarin, a known hepatoprotective agent.

*N. Sativa* extract and some of its components as thymoquinone, dithymoquinone and alpha-hederin have been shown to possess antitumor activities against cancer cell lines, as well as in a diverse animal models of cancer e.g. lung, pancreatic and multiple myeloma [10,40]. Our results confirm such anti-cancer effect for *N. Sativa* in HCC rat model. Although the exact mechanism(s) for the anti-tumor action of *N. Sativa* is not fully understood, it has been postulated that its active components inhibit cell proliferation and arrest cell cycle as well as antioxidant effects [10,41].

Our results clearly show an anti-inflammatory effect of *N. Sativa* as it significantly reduced IL-6 and hs-CRP in our HCC rat model. These results come in agreement with other studies that demonstrated that Thymoquinone, the major *N. Sativa* active component, inhibited the production of IL-6 in human rheumatoid arthritis synovial fibroblast cell culture [42,43]. Another study showed that blockage of IL-6 inhibited mice fibroblast growth factor 19 (FGF19) induced hepatocarcinogenesis [44]. Similarly, activation of IL-6-JAK-STAT3 (signal transducer and activator of transcription 3) signaling pathway accelerated HCC growth in rat model [45]. Since the majority of human HCC is associated with in vivo inflammatory environment [31] *N. Sativa* mediated inhibition/reduction of IL-6 production may contribute to its hepatoprotective effect on HCC. Furthermore, *N. Sativa* reduction of hs-CRP reduction comes in agreement with, Alizadeh et al., 2016 who found that N. *Sativa* reduced hs-CRP in women with rheumatoid arthritis [46]. Along with reduction of IL-6 our results confirm anti-inflammatory effect for *N. Sativa* in HCC as well as the potential role for hs-CRP as an early diagnostic tool for HCC [47].

In addition*, N. Sativa* demonstrated an antioxidant effect in our rat model of HCC as it significantly reduced oxidative stress markers as MDA and increased SOD levels. Studies have shown that both MDA (a byproduct of lipid peroxidation that is produced under oxidative stress conditions) and SOD (a pivotal superoxide scavenging enzyme) are reliable markers of oxidative stress [[48], [49], [50]]. Both MDA and SOD were postulated to be involved in the pathogenesis of HCC [51]. Further, NFE2L2 is a transcription factor that is thought to be involved in hepato-protection against oxidative stress induced HCC mainly by the regulation of many genes involved in glutathione biosynthesis that protect against oxidative damage [52,53]. Together with the aforementioned effect on MDA and SOD, our finding that *N. Sativa* significantly increased NFE2L2 levels in AFB1 induced HCC rat model further supports the idea that its hepatoprotective effect in HCC can be attributed to its antioxidant effect.

*P. Ginseng* and *C*. *Sempervirens* extract administration to our HCC rat model had similar effect to *N. sativa* on anti-inflammatory markers measured (IL-6 and hs-CRP) as well as anti-oxidant markers (MDA, SOD, and NFE2L2). *P. Ginseng* and *C*. *Sempervirens* extract also had hepatoprotective effect in our HCC rat model indicated by histopathological examination and liver enzymes reduction. Our *P. Ginseng* results comes in agreement with other studies that demonstrated hepatoprotective effect for different *P. Ginseng* constituents and extracts against liver cancer in animals [[18], [19], [20], [21]]. It is well documented that *P. Ginseng* extracts and active constituents has anti-inflammatory and antioxidant effects [54,55]. Ginsenoside Rg1, one of *P. Ginseng* components, exhibited a neuroprotective effect via modulation of IL-6 and MDA in rats [56]. Our results further support that this hepatoprotective effect is possibly mediated via reduction of oxidative stress as well as modulation of inflammatory cytokines as IL-6. Our data supports Lu et al., 2016 conclusion who found that Ginseng had hepatoprotective effects against carbon tetrachloride (CCl_4_) liver injury possibly via reduction of liver inflammation [57].

On the other hand, other studies have shown that *C*. *Sempervirens* extract had hepatoprotective effect against CCl_4_ liver injury indicated by liver function tests and liver pathological changes [58,59]. Our results not only concur with these studies but also propose its possible mediation of such hepatoprotective action via anti-inflammatory and anti-oxidant effect. To our knowledge, this is the first demonstration that *C*. *Sempervirens* extract could in fact alter IL-6, hs-CRP together with MDA, SOD, and NFE2L2 AFB1 induced HCC rat model. It was previously reported that *C*. *Sempervirens* extract administration protected against indomethacin-induced gastric ulcer. Such protection was associated with increased SOD activity [26].

Our data supports other studies that showed that health beneficial effects as antioxidant and anti-inflammatory effects can be achieved using plant–derived flavonoids [60,61]. This can be achieved by modulation of gene expression of certain proteins involved in the inflammatory response and alteration of activity levels of enzymes involved in antioxidant response [26].

In conclusion, this study confirms a beneficial hepatoprotective effect for *N. Sativa, P. Ginseng, and C. Sempervirens* extracts orally administered in rat model of AFB1 induced HCC. This effect is putatively mediated via modulation of inflammatory cytokines as IL-6 as well as amelioration of oxidative stress.

## 5 Ethics approval

Animal care and handling was performed in conformance with approved protocols of MSA University and Egyptian Community guidelines for animal care.

## 6 Consent to participate

Not applicable

## 7 Consent to publish

Not applicable

## 8 Availability of data and materials

The datasets supporting the conclusions of this article are included within the article and its additional files.

## 9 Competing interest

The authors declare no competing interest associated with this article. There is no significant financial support for this work that could affect its outcome.

## 10 Author contribution

Dr. Aborehab and Dr. Waly contributed equally to the experimental design, execution of the experiments, data, sample collection the study, conduction of biochemical analyses, interpretation of data. Dr. Waly drafted the manuscript. Dr. Aborehab performed the statistical analyses of this study. Both authors read and approved the final manuscript.

## Funding

This research is not supported by any funding agency.

Transparency document

## References

[1] Ahmed Mohammed H.A. (2017). Factors Influencing Surveillance for Hepatocellular Carcinoma in Patients with Liver Cirrhosis. Liver Cancer.

[2] Siegel R.L., Miller K.D., Jemal A. (2016). Cancer statistics, 2016. CA Cancer J Clin.

[3] Liu Z. (2017). Synergisic effect of APRIL knockdown and Jiedu Xiaozheng Yin, a Chinese medicinal recipe, on the inhibition of hepatocellular carcinoma cell proliferation. Oncol Rep.

[4] Anwar W.A. (2008). Changing pattern of hepatocellular carcinoma (HCC) and its risk factors in Egypt: possibilities for prevention. Mutat Res.

[5] Abdel-Hamid N.M. (2013). *Can methanolic extract of Nigella sativa seed affect glyco-regulatory enzymes in experimental hepatocellular carcinoma?*. Environ Health Prev Med.

[6] Abdel-Aziz F. (2000). Hepatitis C virus (HCV) infection in a community in the Nile Delta: population description and HCV prevalence. Hepatology.

[7] Barakat E.M., El Wakeel L.M., Hagag R.S. (2013). Effects of Nigella sativa on outcome of hepatitis C in Egypt. World J Gastroenterol.

[8] Sun B. (2016). Effect of the herbal formulation Jianpijiedu on the TCRVbetaCDR3 repertoire in rats with hepatocellular carcinoma and subjected to food restriction combined with laxative. Exp Ther Med.

[9] Costa C. (2017). Current evidence on the effect of dietary polyphenols intake on chronic diseases. Food Chem Toxicol.

[10] Dajani E.Z., Shahwan T.G., Dajani N.E. (2016). Overview of the preclinical pharmacological properties of Nigella sativa (black seeds): a complementary drug with historical and clinical significance. J Physiol Pharmacol.

[11] Salem M.L. (2005). *Immunomodulatory and therapeutic properties of the Nigella sativa L. seed.*. Int Immunopharmacol.

[12] Ragheb A. (2009). The protective effect of thymoquinone, an anti-oxidant and anti-inflammatory agent, against renal injury: a review. Saudi J Kidney Dis Transpl.

[13] Attoub S. (2013). Thymoquinone as an anticancer agent: evidence from inhibition of cancer cells viability and invasion in vitro and tumor growth in vivo. Fundam Clin Pharmacol.

[14] Jafri S.H. (2010). Thymoquinone and cisplatin as a therapeutic combination in lung cancer: In vitro and in vivo. J Exp Clin Cancer Res.

[15] Brown R.K. (2014). The effects of thymoquinone and Doxorubicin on leukemia and cardiomyocyte cell lines. Biomed Sci Instrum.

[16] Elsherbiny N.M., El-Sherbiny M. (2014). Thymoquinone attenuates Doxorubicin-induced nephrotoxicity in rats: Role of Nrf2 and NOX4. Chem Biol Interact.

[17] Al-Seeni M.N. (2018). Nigella sativa oil protects against tartrazine toxicity in male rats. Toxicol Rep.

[18] Wang C.Z. (2016). Red ginseng and cancer treatment. Chin J Nat Med.

[19] Kim H.S. (2017). Effect of Red Ginseng on Genotoxicity and Health-Related Quality of Life after Adjuvant Chemotherapy in Patients with Epithelial Ovarian Cancer: A Randomized, Double Blind, Placebo-Controlled Trial. Nutrients.

[20] Zhou B., Wang J., Yan Z. (2014). Ginsenoside Rg3 attenuates hepatoma VEGF overexpression after hepatic artery embolization in an orthotopic transplantation hepatocellular carcinoma rat model. Onco Targets Ther.

[21] Igami K. (2015). Hepatoprotective effect of fermented ginseng and its major constituent compound K in a rat model of paracetamol (acetaminophen)-induced liver injury. J Pharm Pharmacol.

[22] Razgonova M.P. (2019). Panax ginseng components and the pathogenesis of Alzheimer’s disease (Review). Mol Med Rep.

[23] Razgonova M.P. (2019). Supercritical green technologies for obtaining ginsenosides from far-eastern wild ginseng panax ginseng meyer using sfe for applying in drug, food and cosmetic industries. Farmacia.

[24] Bhattacharyya S.S. (2010). Anti-oncogenic potentials of a plant coumarin (7-hydroxy-6-methoxy coumarin) against 7,12-dimethylbenz [a] anthracene-induced skin papilloma in mice: the possible role of several key signal proteins. Zhong Xi Yi Jie He Xue Bao.

[25] Bhattacharyya S.S. (2008). In vitro studies demonstrate anticancer activity of an alkaloid of the plant Gelsemium sempervirens. Exp Biol Med (Maywood).

[26] Koriem K.M., Gad I.B., Nasiry Z.K. (2015). Protective effect of Cupressus sempervirens extract against indomethacin-induced gastric ulcer in rats. Interdiscip Toxicol.

[27] Das R.K., Hossain S.K., Bhattacharya S. (2005). Diphenylmethyl selenocyanate inhibits DMBA-croton oil induced two-stage mouse skin carcinogenesis by inducing apoptosis and inhibiting cutaneous cell proliferation. Cancer Lett.

[28] Takaki A., Yamamoto K. (2015). *Control of oxidative stress in hepatocellular carcinoma: Helpful or harmful?*. World J Hepatol.

[29] Liu H.T. (2016). Effects of coenzyme Q10 supplementation on antioxidant capacity and inflammation in hepatocellular carcinoma patients after surgery: a randomized, placebo-controlled trial. Nutr J.

[30] Maurya B.K., Trigun S.K. (2016). Fisetin Modulates Antioxidant Enzymes and Inflammatory Factors to Inhibit Aflatoxin-B1 Induced Hepatocellular Carcinoma in Rats. Oxid Med Cell Longev.

[31] Ji T. (2016). Distinct role of interleukin-6 and tumor necrosis factor receptor-1 in oval cell- mediated liver regeneration and inflammation-associated hepatocarcinogenesis. Oncotarget.

[32] Ding W.Q. (2004). Differential sensitivity of cancer cells to docosahexaenoic acid-induced cytotoxicity: the potential importance of down-regulation of superoxide dismutase 1 expression. Mol Cancer Ther.

[33] Menegon S., Columbano A., Giordano S. (2016). The Dual Roles of NRF2 in Cancer. Trends Mol Med.

[34] Kim Y.S. (2011). Protective Effect of Korean Red Ginseng against Aflatoxin B1-Induced Hepatotoxicity in Rat. J Ginseng Res.

[35] Rotimi O.A. (2017). Acute aflatoxin B1 - Induced hepatotoxicity alters gene expression and disrupts lipid and lipoprotein metabolism in rats. Toxicol Rep.

[36] Al-Saffar F.J., Ganabadi S., Fakurazi S., Yaakub H., Lip M. (2010). Chondroprotective effect of Zerumbone on Monosodium Iodoacetate induced osteoarthritis in rats. Journal of Applied Sciences.

[37] Dollah M.A. (2013). Toxicity effect of nigella sativa on the liver function of rats. Adv Pharm Bull.

[38] Fu P.P. (2009). Quality assurance and safety of herbal dietary supplements. J Environ Sci Health C Environ Carcinog Ecotoxicol Rev.

[39] Vogler G.A., Suckow M.A., Weisbroth S., Franklin C.L. (2006). Anesthesia and analgesia in the laboratory rat.. Eds.).

[40] Rooney S., Ryan M.F. (2005). Effects of alpha-hederin and thymoquinone, constituents of Nigella sativa, on human cancer cell lines. Anticancer Res.

[41] Raghunandhakumar S. (2013). Thymoquinone inhibits cell proliferation through regulation of G1/S phase cell cycle transition in N-nitrosodiethylamine-induced experimental rat hepatocellular carcinoma. Toxicol Lett.

[42] Umar S. (2012). Modulation of the oxidative stress and inflammatory cytokine response by thymoquinone in the collagen induced arthritis in Wistar rats. Chem Biol Interact.

[43] Umar S. (2015). Thymoquinone inhibits TNF-alpha-induced inflammation and cell adhesion in rheumatoid arthritis synovial fibroblasts by ASK1 regulation. Toxicol Appl Pharmacol.

[44] Zhou M. (2017). Non-cell-autonomous activation of IL-6/STAT3 signaling mediates FGF19-driven hepatocarcinogenesis. Nat Commun.

[45] Hamaguchi Y. (2016). Longer warm ischemia can accelerate tumor growth through the induction of HIF-1alpha and the IL-6-JAK-STAT3 signaling pathway in a rat hepatocellular carcinoma model. J Hepatobiliary Pancreat Sci.

[46] Kheirouri S., Hadi V., Alizadeh M. (2016). Immunomodulatory Effect of Nigella sativa Oil on T Lymphocytes in Patients with Rheumatoid Arthritis. Immunol Invest.

[47] Ma L.N. (2017). Assessment of high-sensitivity C-reactive protein tests for the diagnosis of hepatocellular carcinoma in patients with hepatitis B-associated liver cirrhosis. Oncol Lett.

[48] Deng X.Y. (2015). Cardioprotective effects of timosaponin B II from Anemarrhenae asphodeloides Bge on isoproterenol-induced myocardial infarction in rats. Chem Biol Interact.

[49] Ibrahim S.S., Nassar N.N. (2008). Diallyl sulfide protects against N-nitrosodiethylamine-induced liver tumorigenesis: role of aldose reductase. World J Gastroenterol.

[50] Ungurianu A. (2019). Spectrophotometric versus spectrofluorometric assessment in the study of the relationships between lipid peroxidation and metabolic dysregulation. Chem Biol Drug Des.

[51] Guoyin Z. (2017). Antihepatocarcinoma Effect of Portulaca oleracea L. in Mice by PI3K/Akt/mTOR and Nrf2/HO-1/NF-kappaB Pathway. Evid Based Complement Alternat Med.

[52] Karin M., Dhar D. (2016). Liver carcinogenesis: from naughty chemicals to soothing fat and the surprising role of NRF2. Carcinogenesis.

[53] Suzuki T., Motohashi H., Yamamoto M. (2013). Toward clinical application of the Keap1-Nrf2 pathway. Trends Pharmacol Sci.

[54] Korivi M. (2012). Ginsenoside-Rg1 Protects the Liver against Exhaustive Exercise-Induced Oxidative Stress in Rats. Evid Based Complement Alternat Med.

[55] Smolinski A.T., Pestka J.J. (2003). Modulation of lipopolysaccharide-induced proinflammatory cytokine production in vitro and in vivo by the herbal constituents apigenin (chamomile), ginsenoside Rb(1) (ginseng) and parthenolide (feverfew). Food Chem Toxicol.

[56] Zhang Y. (2016). Neuroprotective effect of ginsenoside Rg1 prevents cognitive impairment induced by isoflurane anesthesia in aged rats via antioxidant, anti-inflammatory and anti-apoptotic effects mediated by the PI3K/AKT/GSK-3beta pathway. Mol Med Rep.

[57] Lu K.H. (2017). Ginseng essence, a medicinal and edible herbal formulation, ameliorates carbon tetrachloride-induced oxidative stress and liver injury in rats. J Ginseng Res.

[58] Ibrahim N.A., El-Seedi H.R., Mohammed M.M. (2007). *Phytochemical investigation and hepatoprotective activity of Cupressus sempervirens L. leaves growing in Egypt*. Nat Prod Res.

[59] Ali S.A. (2010). Protective role of Juniperus phoenicea and Cupressus sempervirens against CCl(4). World J Gastrointest Pharmacol Ther.

[60] Sivaramakrishnan V. (2008). Attenuation of N-nitrosodiethylamine-induced hepatocellular carcinogenesis by a novel flavonol-Morin. Chem Biol Interact.

[61] Moon H.K., Yang E.S., Park J.W. (2006). Protection of peroxynitrite-induced DNA damage by dietary antioxidants. Arch Pharm Res.

